# A retrospective analysis of the relationship between anti-cyclic citrullinated peptide antibody and interstitial lung disease in systemic sclerosis

**DOI:** 10.1038/s41598-022-23180-2

**Published:** 2022-11-10

**Authors:** Jang Woo Ha, Yoo Jin Hong, Hyun Jin Cha, Jeonghun Daniel Moon, Jung Yoon Pyo, Sang-Won Lee, Yong-Beom Park, Chul Hwan Park, Jason Jungsik Song

**Affiliations:** 1 Division of Rheumatology, Department of Internal Medicine, Yonsei University College of Medicine, 50-1 Yonsei-ro, Seodaemun–gu, Seoul, 03722 South Korea; 2 Department of Radiology and Research Institute of Radiological Science, Severance Hospital, Yonsei University College of Medicine, Seoul, Korea; 3 Synapse Center, Yonsei University College of Medicine, Seoul, Korea; 4 Division of Rheumatology, Valley Medical Center, University of Washington Medicine, Renton, WA 98055 USA; 5 Department of Radiology and Research Institute of Radiological Science, Gangnam Severance Hospital, Yonsei University College of Medicine, 211 Eonju-ro, Gangnam-gu, Seoul, 06273 South Korea; 6 Institute for Immunology and Immunological Diseases, Yonsei University College of Medicine, Seoul, Korea

**Keywords:** Immunology, Rheumatology

## Abstract

Anti-cyclic citrullinated peptide antibody testing is used to diagnose rheumatoid arthritis and associated with interstitial lung disease in RA. Herein, we investigate the relationship between anti-CCP antibody and ILD in SSc. We performed a retrospective analysis at a tertiary medical center between 2005 and 2019. Patients with SSc, systemic lupus erythematosus, and polymyositis/dermatomyositis (PM/DM) were evaluated for anti-CCP antibody and ILD. Additionally, medical records of SSc patients with ILD were reviewed. SSc patients had the highest anti-CCP antibody positivity rate compared to those with SLE and PM/DM. The incidence of ILD was higher in SSc patients with anti-CCP antibody than in those without. The usual interstitial pneumonia (UIP) incidence was higher in the anti-CCP antibody-positive group than in the anti-CCP antibody-negative group. The DLCO was lower in the anti-CCP antibody-positive group than in the anti-CCP antibody-negative group. On multivariable analysis, factors associated with SSc-ILD were anti-CCP antibody or rheumatoid factor (β coefficient, 2.652 [95% CI 1.472 to 4.776]) and anti-Scl70 antibody (β coefficient, 4.011 [95% CI 2.142 to 7.508]). Anti-CCP antibody may be associated with a higher incidence of ILD in SSc. SSc patients with anti-CCP antibody may have more UIP pattern and lower DLCO.

*Trial Registration* Retrospectively registered.

## Introduction

Systemic sclerosis (SSc) is a chronic, heterogeneous, multisystem disease characterized by widespread vascular dysfunction with fibrosis of the skin and internal organs, such as the lungs. According to the 2013 Classification Criteria for Systemic Sclerosis, there are three hallmarks of SSc: fibrosis of the skin and/or internal organs, production of specific autoantibodies, and evidence of vasculopathy^1^. Evaluating disease staging and organ involvement is important to guide effective treatment implementation and predict outcomes in SSc. Interstitial lung disease (ILD) is an important internal organ involvement in SSc, which accounts for 33% of deaths occurring in patients with SSc^2^.

Connective tissue disease-associated interstitial lung disease (CTD-ILD) is characterized by inflammation and/or fibrosis of the lungs in patients with systemic rheumatic diseases, such as rheumatoid arthritis (RA), SSc, systemic lupus erythematosus (SLE), and polymyositis/dermatomyositis (PM/DM)^3^. Based on a histopathologic analysis, CTD-ILD can be classified into the following subtypes: nonspecific interstitial pneumonia (NSIP), usual interstitial pneumonia (UIP), organizing pneumonia (OP), apical fibrosis, diffuse alveolar damage (DAD), and lymphoid interstitial pneumonia (LIP)^4^. The two most common subtypes of CTD-ILD are NSIP and UIP^3^. UIP is characterized by non-uniform fibrosis with honeycomb change, fibroblast foci, and mild inflammation, while NSIP is characterized by uniform fibrosis with a varying proportion of interstitial inflammation^4^. The dominant pattern of ILD is UIP in RA, and NSIP in SSc^3^.

For detection and characterization of CTD-ILD, high-resolution computed tomography (HRCT) is more sensitive than chest radiography and conventional computed tomography (CT)^5^. The HRCT pattern is associated with the histopathologic process^6^. A reticular pattern with traction bronchiectasis on HRCT is associated with a fibrotic process, whereas a ground-glass pattern is associated with an inflammatory process^5^. A radiographic and/or histologic pattern in ILD can be a risk factor for progression because the prognosis of UIP is worse than that of non-UIP^7–9^.

Anti-cyclic citrullinated peptide (CCP) antibody is a group of autoantibodies directed against the citrullinated epitopes specific to RA and these appear years before the onset of clinically apparent disease^10^. Anti-CCP antibody is associated with more erosive joint diseases and extra-articular manifestations, such as ILD^10–12^. In a study of 230 RA-ILD patients in the UK, anti-CCP antibody was the strongest predictor of RA-ILD^13^. Interestingly, recent studies have suggested that the anti-CCP antibody positivity rate is increased in patients with SSc^14–18^. However, the role of anti-CCP antibody in SSc-ILD has not been well studied. Therefore, we examined the relationship between anti-CCP antibody and ILD in patients with SSc at our hospital, using HRCT and pulmonary function tests (PFT).

## Methods

### Patient selection and study design

This was a retrospective observational study conducted at a single tertiary care institution (Severance Hospital, Yonsei University Health System, Seoul, South Korea) between January 2005 and May 2021. Patients with lupus, PM/DM, or SSc who had been tested for anti-CCP antibody were included in our study. A total of 936 patients diagnosed with SLE based on the 1997 American College of Rheumatology (ACR) criteria or the 2012 Systemic Lupus Erythematosus International Collaborating Clinics criteria, were included^19,20^. Additionally, 156 patients diagnosed with PM/DM based on the Bohan and Peter criteria, were included^21,22^. Furthermore, 260 patients diagnosed with SSc based on the 2013 ACR/European League Against Rheumatism classification criteria, were included^1^. We further evaluated the diagnosis of RA based on the 2010 ACR classification criteria^23^.

### Ethics approval and consent to participate

This study was approved by the Institutional Review Board of Severance Hospital (IRB approval number: 2021-1213-001) and conducted in accordance with the principles set forth in the Declaration of Helsinki. The requirement for informed consent was waived because of the retrospective nature of the study by the Institutional Review Board of Severance Hospital.

### Assessment of the clinical manifestations and laboratory findings associated with SSc

Clinical manifestations of SSc, including thickening of the skin of the fingers extending to the metacarpophalangeal joints (MCP), puffy fingers, sclerodactyly, fingertip lesions, telangiectasia, Raynaud’s phenomenon, and joint pain, were reviewed. Nail fold capillary abnormalities were assessed by capillary microscopy. The results of anti-nuclear antibody (ANA), anti-Scl-70 antibody, anti-centromere antibody, white blood cells, hemoglobin level, platelets, serum creatinine level, aspartate aminotransferase level, total protein level, albumin level, urine protein-creatinine ratio, right ventricular systolic pressure on echocardiography, and erosive changes in hand radiography were reviewed. These test results were based on the time SSc was diagnosed at our hospital or the time of referral when SSc was diagnosed at another hospital.

### Measurement of anti-CCP antibody

Serum anti-CCP antibody levels were measured using the QUANTA Flash CCP3 (Werfen, San Diego, CA, USA), according to the manufacturer’s instructions. The cut-off point was set to 5 units/mL, and the results of the anti-CCP antibody test were reviewed retrospectively.

### Evaluation of SSc-ILD by HRCT

For patients with SSc-ILD, two chest radiologists (C.H.P. and Y.J.H) with over 10 years of experience in chest CT interpretation, independently analyzed HRCT findings while blinded to the laboratory results and clinical information. HRCT was evaluated according to the guidelines for idiopathic pulmonary fibrosis^24,25^. The presence or absence of honeycombing, traction bronchiectasis, consolidation, ground-glass opacity, and other abnormalities was carefully evaluated, and the distribution of abnormalities was categorized as no abnormality or subpleural, peribronchial, or basal predominance to determine the pattern of ILD. HRCT findings of UIP comprise irregular linear hyperattenuating areas, honeycombing, traction bronchiectasis or bronchiolectasis, architectural distortion, and focal ground-glass attenuation, while HRCT findings of NSIP comprise ground-glass attenuation, irregular linear hyperattenuating areas, and consolidation^5^.

### Evaluation of SSc-ILD by pulmonary function test

PFT results, including forced vital capacity (FVC) and diffusing capacity of the lungs for carbon monoxide (DLCO) at the time of ILD diagnosis in patients with SSc-ILD, were analyzed. DLCO was calculated using the following equation; DLCO predicted corrected = DLCO predicted * (1.7 * hemoglobin/(Age-Sex-Factor + hemoglobin)). For females of any age and children less than 15 years old, the Age-Sex-Factor is 9.38. For males 15 years old or older, the Age-Sex-Factor is 10.22^26^.

### Statistical analysis

Data analysis was conducted using IBM SPSS Statistics for Windows, v. 26 (IBM Corp., Armonk, NY, USA). Continuous variables are presented as medians and inter-quartile ranges, and categorical variables are expressed as frequencies and percentages. Continuous variables were compared using Student’s *t*-test, and categorical data were compared using the Chi-square test or Fisher’s exact test, as appropriate. In all statistical analyses, a two-tailed *p* value of < 0.05 was considered statistically significant. For multiple comparisons problem, *p* values Bonferroni corrected were applied. The risk factors were analyzed by using the univariate and multivariate logistic regression analysis.

## Results

### Association between anti-CCP antibody and ILD in patients with CTD

We evaluated the positivity rate of the anti-CCP antibody and rheumatoid factor (RF) in patients diagnosed with CTD at our hospital. Among patients with SLE, PM/DM, and SSc, those with SSc had the highest incidence of anti-CCP antibody (9.2% vs. 11.5% vs. 16.2%, respectively; *p* = 0.006) while there was no statistical significance in RF single positivity among patients with SLE, PM/DM, and SSc (Table 1). Among 42 SSc patients with anti-CCP antibody, 37 patients had both anti-CCP antibody and RF, while 5 patients had only anti-CCP antibody. In patients with SLE and SSc, the incidence of ILD was higher in the RF single-positive group and the anti-CCP-positive group than in the anti-CCP/RF-negative group (Table 2). In patients with SLE and SSc, the incidence of RA was higher in the anti-CCP-positive group than in the group with RF single positivity (SLE 57.0% vs. 25.2%; *p* < 0.001, SSc 42.9% vs. 16.4%; *p* = 0.006) (see Supplementary Table S1). We also evaluated the relationship between the diagnosis of RA and ILD in CTDs. In patients with SLE, the incidence of ILD was higher in the RA group than in the group not diagnosed with RA (17.4% vs. 6.5%, *p*** < 0.001**) (see Supplementary Table S2). In patients with SSc, there was a trend that the incidence of ILD was higher in the RA group than in the group not diagnosed with RA (65.5% vs. 42.45%, *p* = 0.054), but there was no statistical significance. Additionally, there was no difference in incidence of ILD between anti-CCP-positive SSc patients with RA vs without RA (see Supplementary Table S3).

**Table 1:** Anti-CCP antibody and RF positivity in connective tissue diseases.

	Anti-CCP positive	Anti-CCP/RF double positive	Anti-CCP single positive	RF single positive
SLE (N = 936)	86 (9.2%)	63 (6.7%)	23 (2.5%)	262 (28.0%)
PM/DM (N = 156)	18 (11.5%)	13 (8.3%)	5 (3.2%)	33 (21.2%)
SSc (N = 260)	42 (16.2%)	37 (14.2%)	5 (1.9%)	67 (25.8%)
*p* value	**0.036**	**0.006**	> 0.999	> 0.999

Values are expressed as n (%). All *p* values Bonferroni corrected.

SLE, systemic lupus erythematous; PM, polymyositis; DM, dermatomyositis: SSc, systemic sclerosis.

Significant values are in [bold].

**Table 2:** Relationship between anti-CCP antibody/RF and interstitial lung disease in connective tissue diseases.

	ILD with anti-CCP/RF negative	ILD with RF single positive	ILD with anti-CCP positive	*p* value
SLE	31/588 (5.3%)	28/262 (10.7%)	14/86 (16.3%)	**< 0.001**
PM/DM	41/105 (39.0%)	18/33 (54.5%)	7/18 (38.9%)	0.843
SSc	49/151 (32.5%)	41/67 (61.2%)	27/42 (64.3%)	**< 0.001**

Bold text indicates statistical significance. Values are expressed as n (%). all *p* values Bonferroni corrected.

SLE, systemic lupus erythematous; PM, polymyositis; DM, dermatomyositis; SSc, systemic sclerosis; ILD, interstitial lung disease.

### Baseline characteristics of the patients with SSc based on anti-CCP antibody and RF positivity

We compared baseline clinical characteristics of the patients with SSc based on anti-CCP antibody and RF positivity (Table 3). Abnormal nail fold capillaries were more frequent in the anti-CCP/RF-negative group and the RF single-positive group than in the anti-CCP-positive group. Joint pain and radiographic erosion were more frequent in the anti-CCP-positive group than in the anti-CCP/RF-negative group and the RF single-positive group. Hemoglobin and albumin levels were lower in the anti-CCP-positive group than in the anti-CCP/RF-negative group and the RF single-positive group. Platelet counts and anti-Scl-70 antibody positivity rate was higher in the anti-CCP-positive group than in the anti-CCP/RF-negative group and the RF single-positive group. Although longer disease duration can be associated with ILD severity, there was no difference in disease duration between anti-CCP-positive and -negative groups. Some medications can be confounding factors for ILD severity because steroid, immunosuppressants, and proton pump inhibitors may have a protective effect on ILD progression. In our study, anti-CCP-positive group used steroid, immunosuppressants, and proton pump inhibitors more frequently than did anti-CCP-negative groups (Table 3, see Supplementary Table S4). However, these factors may not increase the risk of ILD because these are not risk factors but protective factors.

**Table 3:** Characteristics of patients with SSc based on anti-CCP antibody and RF positivity.

	Anti-CCP/RF negative (n = 151)	RF single positive (n = 67)	Anti-CCP positive (n = 42)	*p* value
**Demographic data**				
Age at diagnosis (years)	54 (17)	56 (17)	57 (14)	0.274
Male sex	17 (11.3%)	6 (9.0%)	4 (9.5%)	0.859
**Clinical manifestations**				
Thickening of the skin on the fingers, extending to the MCP joints	42 (27.8%)	18 (26.9%)	14 (33.3%)	0.740
Puffy fingers	18 (11.9%)	4 (6.0%)	4 (9.5%)	0.400
Sclerodactyly	96 (63.6%)	45 (67.2%)	28 (66.7%)	0.851
Fingertip lesions	38 (25.2%)	16 (23.9%)	8 (19%)	0.714
Telangiectasia	9 (6.0%)	1 (1.5%)	4 (9.5%)	0.175
Abnormal nailfold capillaries	44 (29.1%)	16 (23.9%)	4 (9.5%)	**0.033**
Pulmonary arterial hypertension	28 (18.7%)	16 (23.9%)	13 (31.0%)	0.217
Raynaud’s phenomenon	111 (73.5%)	47 (70.1%)	28 (66.7%)	0.658
Joint pain	60 (39.7%)	29 (43.3%)	29 (69.0%)	**0.003**
X-ray erosion	7/67 (10.4%)	5/34 (14.7%)	10/33 (30.3%)	**0.041**
Smoking history	12/86 (14.0%)	5/41 (12.2%)	5/28 (17.9%)	0.801
**Autoantibodies**				
Anti-centromere	61/150 (40.7%)	20/64 (31.3%)	10/41 (24.4%)	0.109
Anti-Scl-70	40/150 (26.7%)	20/64 (31.3%)	21/41 (51.2%)	**0.011**
ANA	141/148 (95.3%)	64/65 (98.5%)	41/41 (100.0%)	0.214
**Echocardiography findings**				
RVSP (mmHg)	28.0 (14.5)	30.0 (14.0)	34.0 (17.0)	0.163
**Laboratory findings**				
White blood cell count (/mm^3^)	6505 (4220)	6840 (6350)	10,310 (5650)	0.073
Hemoglobin (g/dL)	13.0 (2.1)	12.6 (1.5)	12.2 (1.9)	**0.008**
Platelet count (10^3^/mm^3^)	251 (94)	200 (106)	318 (111)	** < 0.001**
Serum creatinine (mg/dL)	0.7 (0.2)	0.7 (0.2)	0.7 (0.2)	0.660
Aspartate aminotransferase (IU/L)	21 (9)	23 (14)	20 (21)	0.297
Total protein (g/dL)	7.1 (0.7)	7.1 (0.4)	6.7 (1.0)	0.086
Serum albumin (g/dL)	4.3 (0.5)	4.1 (0.6)	3.8 (0.7)	** < 0.001**
Urine protein-creatinine ratio	0.1 (0.1)	0.1 (0.2)	0.1 (0.2)	0.057
**Progress**				
Follow up duration (month)	40.0 (55.3)	42.0 (46.0)	45.0 (128.0)	0.948
**Medication**				
Steroid	58 (38.4%)	38 (56.7%)	33 (78.6%)	**< 0.001**
Immunosuppressants	67 (44.4%)	28 (41.8%)	30 (71.4%)	**0.004**
Vasodilator	87 (57.6%)	38 (56.7%)	29 (69.0%)	0.367
Anti-fibrotics	33 (21.9%)	13 (19.4%)	10 (23.8%)	0.854
PPI	52 (34.4%)	29 (43.3%)	23 (54.8%)	**0.049**

Bold text indicates statistical significance. Values are expressed as medians (interquartile range, IQR) or n (%).

SSc, systemic sclerosis; ILD, interstitial lung disease; MCP, metacarpophalangeal; ANA, antinuclear antibody; RVSP, right ventricular systolic pressure; PPI, proton pump inhibitor.

### Comparison of HRCT pattern based on anti-CCP antibody in patients with SSc-ILD

**Figure 1:**
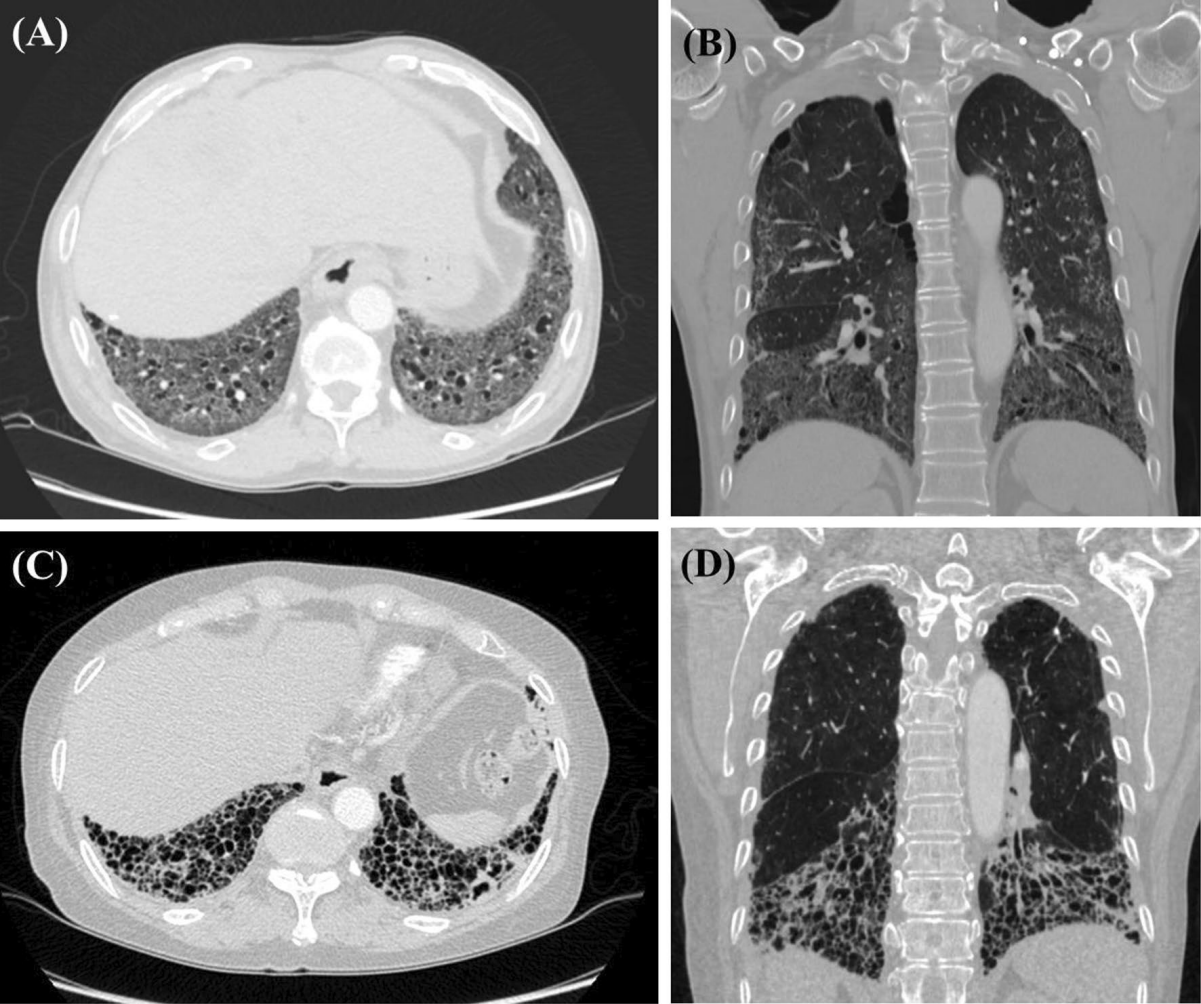
Representative HRCT images of patients with SSc-ILD. Axial (**A**) and coronal (**B**) HRCT images of an anti-CCP antibody-negative patient with SSc-ILD. They show extensive ground-glass opacities and traction bronchiectasis with basal predominance. These findings are typical of a non-specific interstitial pneumonia pattern. Axial (**C**) and coronal (**D**) HRCT images of an anti-CCP antibody-positive patient with SSc-ILD. They show predominant lower lobe pulmonary fibrosis and traction bronchiectasis with exuberant honeycombing sign. These findings are consistent with the usual interstitial pneumonia pattern. HRCT: high resolution computed tomography; SSc: systemic sclerosis; ILD: interstitial lung disease.

The anti-CCP-positive group had a higher UIP incidence than the anti-CCP-negative group (55.6% vs. 31.1%, *p* = 0.021) (Table 4). RF single, anti-Scl-70 and anti-centromere antibodies were not associated with UIP in patients with SSc-ILD. The anti-CCP-positive group had a lower incidence of ground-glass opacities than the anti-CCP -negative group (63.0% vs. 84.4%, *p* = 0.015) (see Supplementary Table S5. Figure 1A,B show the representative NSIP pattern in anti-CCP-negative SSc patients. They also show extensive ground-glass opacities and traction bronchiectasis, with basal predominance. Figure 1C,D show the representative UIP pattern in anti-CCP -positive SSc patients. They also show predominant lower lobe pulmonary fibrosis and traction bronchiectasis with exuberant honeycombing sign.

**Table 4:** Pattern of ILD in SSc patients based on the presence of autoantibodies.

	Autoantibody positive	Autoantibody negative	*p* value
**Anti-CCP antibody**			
UIP	15/27 (55.6%)	28 /90 (31.1%)	**0.021**
Non-UIP (NSIP, OP)	12/27 (44.4%)	62/90 (68.9%)	
**RF single**			
UIP	18/41 (43.9%)	25/76 (32.9%)	0.239
Non-UIP (NSIP, OP)	23/41 (56.1%)	51/76 (67.1%)	
**Anti-Scl-70 antibody**			
UIP	19/56 (33.9%)	24/61 (39.3%)	0.544
Non-UIP (NSIP, OP)	37/56 (66.1%)	37/61 (60.7%)	
**Anti-centromere antibody**			
UIP	5/12 (41.7%)	35/101 (34.7%)	0.751
Non-UIP (NSIP, OP)	7/12 (58.3%)	66/101 (65.3%)	

Bold text indicates statistical significance. Values are expressed as N (%).

UIP, usual interstitial pneumonia; NSIP, non-specific interstitial pneumonia; OP, organizing pneumonia; SSc, systemic sclerosis; ILD, interstitial lung disease.

### Comparison of PFT results based on anti-CCP antibody and RF positivity in patients with SSc-ILD

The PFT results demonstrated that the DLCO was lower in the anti-CCP antibody-positive group than in the anti-CCP antibody-negative group (57.2% vs. 67.8%, respectively; *p* = 0.006) (Table 5). Because DLCO is low in anti-CCP-positive patients, we further evaluate the presence of PAH. In SSc patients, DLCO was lower in patients with PAH than in patients without PAH (see Supplementary Table S6). However, there is no difference of RVSP between anti-CCP-positive vs. anti-CCP-negative groups with low DLCO (see Supplementary Table S7). There was no significant difference in the FVC between the anti-CCP-positive and -negative groups. Furthermore, there was no significant difference in the DLCO or FVC between the antibody-positive and -negative groups for other autoantibodies such as the RF single, anti-Scl-70, and anti-centromere antibodies.

**Table 5:** Effect of autoantibodies on pulmonary function in SSc-ILD.

	Autoantibody positivity	Autoantibody negativity	*p* value
**Anti-CCP antibody**			
FVC (%)	70.5 (24.0)	73.0 (26.0)	0.518
DLCO (%)	57.2 (31.9)	67.8 (31.3)	**0.006**
**RF single**			
FVC (%)	67.0 (25.0)	72.5 (27.0)	0.438
DLCO (%)	63.4 (30.7)	59.6 (30.2)	0.933
**Anti-Scl-70 antibody**			
FVC (%)	70.0 (20)	72.0 (27.0)	0.142
DLCO (%)	62.4 (31.0)	60.8 (34.6)	0.197
**Anti-centromere antibody**			
FVC (%)	72.0 (23.0)	70.5 (27.0)	0.693
DLCO (%)	69.4 (49.7)	61.0 (30.6)	0.703

Bold text indicates statistical significance. Values are expressed as median (IQR).

PFT, pulmonary function test; FVC, forced volume capacity; DLCO, diffusing lung capacity of lung for CO; SSc, systemic sclerosis; ILD, interstitial lung disease.

### Univariate and multivariate regression analysis for identification of biomarkers for ILD in SSc

To further validate the association between ILD and anti-CCP/RF, we performed univariate and multivariate regression analysis. We compared several factors associated with arthritis phenotypes such as anti-CCP antibody, RF, diagnosis of RA, joint pain, and joint erosion. Anti-CCP antibody or RF positivity is the most statistically significant when univariate regression analysis was performed (Table 6). Therefore, we used anti-CCP antibody or RF positivity for multivariate regression analysis. Other parameters such as abnormal nailfold capillaries, anti-Scl70 antibody, white blood cell count, platelet, and albumin were significant in univariate regression analysis and were used for multivariate regression analysis. When multivariate regression analysis on factors associated with ILD was performed, anti-CCP antibody or RF, anti-Scl70 antibody, white blood cell count, and albumin were statistically significant (Table 7). The association between anti-Scl70 antibody and ILD has been well known, while the association between anti-CCP antibody or RF and ILD in SSc has not been addressed before.

**Table 6:** Univariate linear regression analysis for ILD in patients with SSc.

	Beta coefficient (95%CI)	*p* value
Anti-CCP	2.560 (1.289, 5.085)	**0.007**
RF single	2.428 (1.373, 4.292)	**0.002**
Anti-CCP or RF	3.452 (2.061, 5.784)	**< 0.001**
RA overlap	2.579 (1.148, 5.790)	**0.022**
Joint pain	1.360 (0.832, 2.223)	0.220
X-ray erosion	1.385 (0.553, 3.466)	0.487
Abnormal nailfold capillaries	0.383(0.208, 0.707)	**0.002**
Anti-Scl70 antibody	4.598 (2.606, 8.113)	**< 0.001**
Platelet count	1.006 (1.002, 1.009)	**0.001**
Albumin	0.224 (0.118, 0.425)	**< 0.001**
White blood cell count	1.255 (1.136, 1.387)	** < 0.001**
Hemoglobin	0.952 (0.812, 1.117)	0.550

Bold text indicates statistical significance.

SSc, systemic sclerosis; ILD, interstitial lung disease; RA, rheumatoid arthritis.

**Table 7:** Factors associated with ILD in patients with SSc in multivariate linear regression analysis.

	Beta coefficient (95%CI)	*p* value
Anti-CCP antibody or RF	2.652 (1.472, 4.776)	**0.001**
Anti-Scl70 antibody	4.011 (2.142, 7.508)	**< 0.001**
White blood cell count	1.005 (1.001, 1.008)	**0.002**
Albumin	0.301 (0.150, 0.605)	**0.001**

Bold text indicates statistical significance.

ILD, interstitial lung disease.

## Discussion

Although the anti-CCP antibody is highly specific for RA, it has been reported to be positive in other diseases, such as tuberculosis (32%), SLE (17%), psoriatic arthritis (15.6%), autoimmune hepatitis (9%), idiopathic pulmonary fibrosis (6.5%), and primary biliary cholangitis (2.7%)^27–31^. Indeed, the anti-CCP antibody positivity rate is higher in patients with CTDs, including SLE, PM/DM, and SSc, than in the general population^32^. Interestingly, our results demonstrated that the anti-CCP antibody positivity rate was higher in patients with SSc (16.2%) than in those with SLE (9.2%) or PM/DM (11.5%), using data from a single tertiary center. We also observed that anti-CCP antibody positivity was associated with a higher incidence of ILD in SSc. Our observation is consistent with that of a previous meta-analysis, which showed that anti-CCP antibody may be associated with pulmonary fibrosis in patients with SSc^33^.

We further evaluated the subtype and severity of ILD using HRCT and PFT in SSc patients, based on anti-CCP antibody positivity. Among patients with SSc-ILD, the anti-CCP antibody-positive group had a predominantly UIP pattern, while the anti-CCP antibody-negative group had a predominantly NSIP pattern. Furthermore, SSc patients with anti-CCP antibody demonstrated a lower DLCO. This is consistent with the results of a study on idiopathic pulmonary fibrosis, which found that UIP was less reversible and had a worse prognosis than NSIP^24,34,35^. In our study, anti-CCP antibody positivity is associated with lower frequency of ground-glass opacities in HRCT. Therefore, it may suggest that anti-CCP antibody positivity is associated with poor response to treatment. Alternatively, it may be the result of a delay in detection of ILD which can be associated with more fibrotic changes. However, there was no difference in disease duration between anti-CCP-positive and -negative groups. Therefore, the anti-CCP antibody might be helpful to predict the prognosis of ILD in SSc patients. It is unknown whether the anti-CCP antibody plays a pathologic role in CTD-ILD or is merely a biomarker associated with RA. Interestingly, our data demonstrated an association between anti-CCP antibody positivity and ILD in patients with SSc and SLE. It is also known that anti-CCP antibody positivity is associated with CTD-ILD in patients with anti-synthetase syndrome^36^. Therefore, the anti-CCP antibody could have played a role not only in RA but also in CTD-ILD.

In the disease progression of RA, the appearance of the anti-CCP antibody precedes the symptoms of arthritis^37^. This suggests that anti-CCP antibodies may occur outside the joints. Indeed, the anti-CCP antibody was found in the sputum of asymptomatic first-degree relatives of patients with RA^38^. Furthermore, citrullination of proteins has been found in the lungs of patients with early RA, and the anti-CCP antibody was observed in their bronchoalveolar lavage fluid^39^. It is possible that lung inflammation plays a role in the development of the anti-CCP antibody by inducing peptidylarginine deiminase to produce citrullinated epitopes^40^. The anti-CCP antibody was also detected in non-RA patients with UIP^41^. The presence of subclinical HRCT abnormalities has been reported in anti-CCP antibody positive patients without arthritis^42^. Further studies are needed to determine the role that anti-CCP antibody plays in CTD-ILD.

Alternatively, these findings may suggest that there are overlap syndromes with features of both RA and other CTDs, such as SSc, SLE, or PM/DM^43^. The term “overlap syndrome” has been used if anti-CCP antibodies are present in patients with non-RA rheumatologic disorder. The presence of the anti-CCP antibody appears to be a good marker for joint synovitis and erosive changes in patients with SSc-RA overlap syndrome^30^. In our study, radiographic evidence of erosive change and joint pain was also more frequently observed in SSc patients with the anti-CCP antibody than in SSc patients without the anti-CCP antibody. However, radiography detected join erosion in only 30% of anti-CCP positive SSc patients. Furthermore, based on the ACR classification criteria for RA, 42.9% of anti-CCP positive SSc patients can be classified as RA overlap syndrome. Although anti-CCP antibody positivity is associated with RA overlap syndrome in SSc, more than 50% of anti-CCP-positive SSc patients did not have clear features of RA in our study population. When anti-CCP antibody is positive in SSc patients, it is important to monitor not only joint symptoms but also development of ILD.

It is interesting to find that RF is also associated with ILD in SSc. In our study, 25.8% of SSc patients were RF single positive. The pattern of ILD is different between RF single positivity and anti-CCP antibody positivity. RF single positivity is more frequent in non-UIP while anti-CCP antibody is more frequent in UIP. Our data demonstrated that anti-CCP antibody or RF is associated with ILD in SSc independent of anti-Scl70 antibodies. Therefore, it will be helpful to evaluate anti-CCP antibody or RF in SSc for the early detection of ILD.

This study has several limitations. First, the data for this study were collected retrospectively; this may have led to bias in patient selection and analysis. Second, since this was an observational, cross-sectional study, the long-term prognostic outcomes were not evaluated. Recently, several immunosuppressants such as mycophenolate mofetil, cyclophosphamide, rituximab, abatacept, and calcineurin inhibitors demonstrated therapeutic effects in CTD-ILD. Therefore, it is important to identify serologic markers to guide treatment strategies for SSc-ILD. Further research is needed to evaluate treatment response according to anti-CCP antibody positivity in SSc. Third, a variety of cofounding factors can affect the frequency of anti-CCP antibody and ILD in SSc. However, some of them are lacking due to the retrospective nature of the study such as the modified Rodnan skin score or the types of skin involvement (limited vs. diffuse). Therefore, we can only draw modest conclusion.

## Conclusions

Anti-CCP antibody may be associated with a higher incidence of ILD in SSc. SSc patients with anti-CCP antibody may have more UIP pattern and lower DLCO. An anti-CCP antibody test may be helpful for early detection of ILD in SSc patients.

## Supplementary Information

Supplementary Information.

## Data Availability

The datasets supporting the conclusions of this article are included within the article and its additional supporting files.

## Abbreviations

CTD-ILD: Connective tissue disease-associated interstitial lung disease
PM: Polymyositis
DM: Dermatomyositis
NSIP: Nonspecific interstitial pneumonia
UIP: Usual interstitial pneumonia
OP: Organizing pneumonia
HRCT: High-resolution computed tomography
CT: Computed tomography
PAH: Pulmonary arterial hypertension
SSc: Systemic sclerosis
RA: Rheumatoid arthritis
SLE: Systemic lupus erythematosus
ACR: American College of Rheumatology
PFT: Pulmonary function test
FVC: Forced vital capacity
DLCO: Diffusing capacity of the lung for carbon monoxide
SLE: Systemic lupus erythematous
