# Supplementary material for: A retrospective analysis of the relationship between anti-cyclic citrullinated peptide antibody and interstitial lung disease in systemic sclerosis
Source: Sci Rep. 2022 Nov 10;12:19253. doi: 10.1038/s41598-022-23180-2 (PMC9649731; doi:10.1038/s41598-022-23180-2)
Supplement: Supplementary file 1 — Supplementary Information. [file 41598_2022_23180_MOESM1_ESM.docx]

Supplementary Table S1. Incidence of rheumatoid arthritis overlap syndrome based on anti-CCP antibody and RF in connective tissue diseases

|  | RA with RF single positivity | RA with anti-CCP positivity | P-value |
| --- | --- | --- | --- |
| SLE | 66/262 (25.2%) | 49/86 (57.0%) | **<0.001** |
| PM/DM | 12/33 (36.4%) | 13/18 (72.7%) | 0.060 |
| SSc | 11/67 (16.4%) | 18/42 (42.9%) | **0.006** |

Bold text indicates statistical significance. Values are expressed as n (%). all p-values Bonferroni corrected

SLE, systemic lupus erythematous; PM, polymyositis; DM, dermatomyositis; SSc, systemic sclerosis; RA, rheumatoid arthritis

Supplementary Table S2. Relationship between diagnosis of rheumatoid arthritis and interstitial lung disease in connective tissue diseases

|  | ILD with non-RA | ILD with RA | P-value |
| --- | --- | --- | --- |
| SLE | 53/821 (6.5%) | 20/115 (17.4%) | **<0.001** |
| PM/DM | 55/131 (42.0%) | 11/25 (44.0%) | >0.999 |
| SSc | 98/231 (42.4%) | 19/29 (65.5%) | 0.054 |

Bold text indicates statistical significance. Values are expressed as n (%). all p-values Bonferroni corrected

SLE, systemic lupus erythematous; PM, polymyositis; DM, dermatomyositis; SSc, systemic sclerosis; RA, rheumatoid arthritis; ILD, interstitial lung disease

Supplementary Table S3. Incidence of ILD based on diagnosis of RA and anti-CCP antibody positivity

|  | anti-CCP/RF (-) SSc without RA | anti-CCP (+) SSc  with RA* | anti-CCP (+) SSc without RA* | P-value |
| --- | --- | --- | --- | --- |
| ILD | 49/151 (32.5%) | 12/18 (66.7%) | 15/24 (62.5%) | **<0.001** |

Bold text indicates statistical significance. Values are expressed as n (%).

* Chi-square test for anti-CCP (+) SSc with RA vs. without RA, P = 0.780

SSc, systemic sclerosis; ILD, interstitial lung disease

Supplementary Table S4. Immunosuppressant use of patients with SSc based on anti-CCP antibody and RF positivity

|  | Anti-CCP/RF negative (n=151) | RF single positive (n=67) | Anti-CCP positive (n=42) | P-value |
| --- | --- | --- | --- | --- |
| Immunosuppressant |  |  |  |  |
| Mycophenolate mofetil | 14 (9.3%) | 5 (7.5%) | 5 (11.9%) | 0.738 |
| Calcineurin inhibitor | 6 (4.0%) | 3 (4.5%) | 6 (14.3%) | **0.035** |
| Azathioprine | 13 (8.6%) | 6 (9.0%) | 3 (7.1%) | 0.942 |
| Rituximab | 0 (0%) | 0 (0%) | 1 (2.4%) | 0.075 |
| Cyclophosphamide | 2 (1.3%) | 0 (0%) | 0 (0%) | 0.484 |
| Leflunomide | 1 (0.7%) | 1 (1.5%) | 3 (7.1%) | **0.025** |
| Methotrexate | 17 (11.3%) | 3 (4.5%) | 4 (9.5%) | 0.281 |
| Hydroxychloroquine | 11 (7.3%) | 10 (14.9%) | 4 (9.5%) | 0.212 |

Bold text indicates statistical significance. Values are expressed as n (%).

SSc, systemic sclerosis

Supplementary Table S5. Findings of ILD on HRCT in patients with SSc based on the presence of autoantibodies

|  | Autoantibody positivity | Autoantibody negativity | P-value |
| --- | --- | --- | --- |
| **Anti-CCP antibody** |  |  |  |
| Honeycombing | 14/27 (51.9%) | 29/90 (32.2%) | 0.064 |
| Traction bronchiectasis | 24/27 (88.9%) | 68/90 (75.6%) | 0.184 |
| Ground-glass opacities | 17/27 (63.0%) | 76/90 (84.4%) | **0.015** |
| Subpleural predominance | 25/27 (92.6%) | 82/90 (91.1%) | >0.999 |
| Peribronchial predominance | 12/27 (44.4%) | 46/90 (51.1%) | 0.543 |
| Basal predominance | 24/27 (88.9%) | 83/90 (92.2%) | 0.695 |
| **RF single** |  |  |  |
| Honeycombing | 16/41 (39.0%) | 24/76 (31.6%) | 0.418 |
| Traction bronchiectasis | 36/41 (87.8%) | 64/76 (84.2%) | 0.785 |
| Ground-glass opacities | 32/41 (78.0%) | 60/76 (78.9%) | 0.910 |
| Subpleural predominance | 36/41 (87.8%) | 69/76 (90.8%) | 0.751 |
| Peribronchial predominance | 18/41 (43.9%) | 36/76 (47.4%) | 0.720 |
| Basal predominance | 39/41 (95.1%) | 68/76 (89.5%) | 0.491 |
| **Anti-Scl-70 antibody** |  |  |  |
| Honeycombing | 18/56 (32.1%) | 25/61 (41.0%) | 0.322 |
| Traction bronchiectasis | 44/56 (78.6%) | 48/61 (78.7%) | 0.988 |
| Ground-glass opacities | 43/56 (76.8%) | 50/61 (82.0%) | 0.488 |
| Subpleural predominance | 51/56 (91.1%) | 56/61 (91.8%) | >0.999 |
| Peribronchial predominance | 26/56 (46.4%) | 32/61 (52.5%) | 0.515 |
| Basal predominance | 52/56 (92.9%) | 55/61 (90.2%) | 0.745 |
| **Anti-centromere antibody** |  |  |  |
| Honeycombing | 6/12 (50.0%) | 34/101 (33.7%) | 0.340 |
| Traction bronchiectasis | 7/12 (58.3%) | 82/101 (81.2%) | 0.126 |
| Ground-glass opacities | 9/12 (75.0%) | 81/101 (80.2%) | 0.707 |
| Subpleural predominance | 12/12 (100%) | 91/101 (90.1%) | 0.596 |
| Peribronchial predominance | 6/12 (50.0%) | 50/101 (49.5%) | >0.999 |
| Basal predominance | 11/12 (91.7%) | 93/101 (92.1%) | >0.999 |

Bold text indicates statistical significance. Values are expressed as n (%).

HRCT, high resolution computed tomography; SSc, systemic sclerosis; ILD, interstitial lung disease

Supplementary Table S6. DLCO in SSc patients with PAH

|  | Without PAH (N=68) | With PAH (N=28) | P-value |
| --- | --- | --- | --- |
| DLCO | 67.8 (28.8) | 45.3 (34.0) | **<0.001** |
|  |  |  |  |

Values are expressed as medians (interquartile range, IQR). SSc, systemic sclerosis; PAH, pulmonary arterial hypertension; DLCO, diffusing lung capacity of lung for CO

Supplementary Table S7. RVSP in patients with SSc based on anti-CCP antibody and RF positivity

|  | Anti-CCP/RF negative (n=41) | RF single positive (n=33) | Anti-CCP positive (n=22) | P-value |
| --- | --- | --- | --- | --- |
| RVSP (mmHg) |  |  |  |  |
| Normal DLCO group | 27.0 (5.8) | 26.0 (11.5) | 28.0 (4.5) | 0.367 |
| Low DLCO group | 40.0 (18.3) | 36.0 (34.0) | 42.7 (24.0) | 0.720 |

Values are expressed as medians (interquartile range, IQR)

SSc, systemic sclerosis; RVSP, right ventricular systolic pressure
